# FTO Alleviates Hepatic Ischemia-Reperfusion Injury by Regulating Apoptosis and Autophagy

**DOI:** 10.1155/grp/5587859

**Published:** 2025-01-06

**Authors:** Pi-Xiao Wang, Ling Zhu, Mei Xiang, Rixin Zhang, Xiaolin Zheng, Zhi Zheng, Kai Li

**Affiliations:** ^1^Department of Hepatobiliary and Pancreatic Surgery, The Central Hospital of Wuhan, Tongji Medical College, Huazhong University of Science and Technology, Wuhan, China; ^2^Department of Cardiology, The Central Hospital of Wuhan, Tongji Medical College, Huazhong University of Science and Technology, Wuhan, China

**Keywords:** apoptosis, autophagy, FTO, ischemia-reperfusion injury

## Abstract

**Objective:** Despite N^6^-methyladenosine (m^6^A) being closely involved in various pathophysiological processes, its potential role in liver injury is largely unknown. We designed the current research to study the potential role of fat mass and obesity-associated protein (FTO), an m^6^A demethylase, on hepatic ischemia-reperfusion injury (IRI).

**Methods:** Wild-type mice injected with an adeno-associated virus carrying fat mass and obesity-associated protein (AAV-FTO) or adeno-associated virus carrying green fluorescent protein (GFP) (AAV-GFP) were subjected to a hepatic IRI model in vivo. Hematoxylin–eosin staining was performed to observe IRI. Terminal deoxynucleotidyl transferase dUTP nick end labeling (TUNEL) staining was used to observe the cell apoptosis. Reverse transcription polymerase chain reaction (RT-PCR) was used to observe the expression of FTO. The protein levels of FTO, apoptosis, or autophagy-associated signaling proteins were detected by western blot. Reactive oxygen species (ROS) levels were determined by flow cytometry, and immunohistochemistry was used to detect the FTO and LC3-II expression. For in vitro experiments, cultured hepatocytes were subjected to hypoxia/reoxygenation (H/R) stimulation. Monodansylcadaverine (MDC) staining was used to visualize autophagic vesicles.

**Results:** In the present study, we showed that FTO was involved in hepatic IRI, apoptosis, and autophagy. Specifically, the expression level of FTO was significantly reduced in the hepatic IRI. Besides, increasing FTO expression (AAV-FTO) ameliorated the hepatic IRI in animal models, accompanied by decreased apoptosis and autophagy. Furthermore, the FTO inhibitor (FB23-2) aggravated autophagy in hepatocytes upon H/R-induced damage.

**Conclusion:** FTO could act as a protective effector during hepatic IRI, associated with decreased apoptosis and autophagy. FTO-mediated m^6^A demethylation modification may be an important therapeutic target for hepatic IRI.

## 1. Introduction

Hepatic ischemia-reperfusion injury (IRI) is a significant cause of severe complications or even death during the perioperative period, which is usually accompanied by massive liver cell damage and dysfunction of liver cell regeneration, resulting in liver function impairment or even liver failure [1]. At present, it is still lacking effective measures to alleviate liver IRI [2]. Therefore, it is of great scientific significance and clinical value to search for effective targets that can alleviate hepatocyte death and promote hepatocyte repair and regeneration during IRI.

Hepatic IRI is characterized by tissue hypoxia, induced oxidative stress, and immune response due to limited blood supply and reperfusion (Rep). N^6^-methyladenosine (m^6^A) is the most common internal mRNA modification in eukaryotes, affecting a variety of biological processes [3, 4], and fat mass and obesity-associated protein (FTO) is the first identified RNA demethylase that reverses m^6^A modification [5]. Studies have found that FTO can protect liver IRI by destroying DRP1-mediated mitochondrial debris [6]. However, whether FTO can affect reactive oxygen species (ROS)–mediated mitochondrial autophagy and cell apoptosis in the process of liver IRI has not been reported. In this study, we first constructed an animal model of liver IRI to evaluate the changes of FTO expression at different time points of ischemia and Rep. Besides, we constructed adenovirus overexpression vectors to further study the effects of FTO on ROS content and autophagy and further explore its underlying mechanism.

## 2. Materials and Methods

### 2.1. Animals

Forty-two male C57BL/6 mice (6–8 weeks, 24–26 g) were used as wild-type mice in this study. All of the animals were housed in a specific pathogen-free environment, with controlled appropriate temperature and a 12-h light/dark photocycle. All mice were available ad libitum with fodder and water. The mice were randomly divided into six groups (*n* = 5 per group): control, ischemia-hypoxia (Isch), and Rep for different periods. Adeno-associated virus (AAV)–mediated gene overexpression in the liver of mice was performed as described in our recently published study [7]. To specifically overexpress FTO in the liver adeno-associated virus carrying fat mass and obesity-associated protein (AAV-FTO), an AAV vector (Serotype 8) carrying FTO coding sequence (NM_011936.2) under the control of the thyroxin-binding globulin (TBG) promoter was used (*n* = 6). For the control group, adeno-associated virus carrying green fluorescent protein (GFP) (AAV-GFP) was used (*n* = 6). The expression vectors mediated gene expression in the liver of mice that was achieved via tail vein injection (2 × 10^12^ viral genomes per mouse in 100 *μ*L saline). Four weeks after the virus injection, the operation of the ischemia-reperfusion (I/R) injury model was performed. Detailed methods have been described in our previous study [8]. All animal experiments were approved by the Experimental Animal Ethics Committee of Tongji Medical College, Huazhong University of Science and Technology.

### 2.2. Liver I/R Model

The liver I/R model was constructed according to the methods reported before [9]. Experimental mice were anesthetized by intraperitoneal injection of pentobarbital sodium (50 mg/kg, Sigma-Aldrich, P3761). The liver IRI model was established in all animals except for those in the control group. The hepatic portal vein and hepatic artery were separated after anesthesia in C57BL/6 mice. Then a noninvasive clamp was applied. When the liver lobe turned white, it indicated that the occlusion was successful. After continuous ischemia for 60 min, the vascular clip was removed to restore blood flow Rep in the ischemic liver, and the abdominal cavity was sutured. At the end of Rep, mice were sacrificed. Meanwhile, the blood and ischemic liver lobes were collected for further analysis.

### 2.3. m^6^A Measurements

The m^6^A contents in total RNA were measured by using an m^6^A RNA Methylation Assay Kit (#ab185912, Abcam). Firstly, total RNA was isolated from liver tissues. Then, a 200-ng RNA was coated on strip wells using an RNA high-binding solution. Next, each well was washed and incubated with capture and detection antibodies. The detected signal of m^6^A was enhanced by an enhancer antibody and color developing solution. The signal results were obtained in a microplate reader at a wavelength of 450 nm. The m^6^A levels were calculated from the results of colorimetric analysis.

### 2.4. Hematoxylin–Eosin (HE) Staining

Liver tissues were soaked in 10% neutral formalin. Then, the tissues were mounted in paraffin wax. The paraffin-embedded samples were sliced at 5 *μ*m. For pathological staining, after xylene dewaxing and gradient ethanol hydration, the sections were stained with HE. Then, the sections were mounted with neutral glue. The pictures of the liver tissues were observed and captured using a modular digital microscope system (MD1000, Leica, Solms, Germany).

### 2.5. Terminal Deoxynucleotidyl Transferase dUTP Nick End Labeling (TUNEL) Staining

The paraffin-embedded liver tissues were stained by using the Apoptosis Fluorescein Detection Kit (#S7111, Millipore, Burlington, MA, United States) to evaluate the apoptotic cells. Briefly, the sections were dewaxed and dehydrated in gradient ethanol and then covered with 100 *μ*L of protease K (20 *μ*g/mL) for 20 min. After washing three times with phosphate buffered saline (PBS), slices were soaked in equilibrium buffer for 10 min and then incubated with TDT reaction mixture in the dark at 37°C for 1 h. Finally, 4⁣′,6-diamidino-2-phenylindole (DAPI) was stained to label the nucleus.

### 2.6. Immunohistochemistry

Liver tissue sections were deparaffinized and rehydrated before being subjected to antigen retrieval in a microwave oven. The sections were incubated overnight with primary antibodies against LC3-II (1:100, NB100-2220SS, Novus) or FTO (1:1000, 27226-1-AP, Proteintech) at 4°C. Then, the sections were stained with secondary antibodies by using an immunohistochemistry kit (MaxVision, kit-5020, MXB Biotechnologies, Fuzhou, China). Harris' hematoxylin was used for further counterstaining. The pictures were visualized and captured under a light microscope (Olympus, Tokyo, Japan).

### 2.7. ROS Detection

Liver tissue was grinded and suspended in PBS. Cell suspension was collected for measuring ROS production by using the ROS Assay Kit (Beyotime, S0033M). Firstly, the cells were suspended with 10 mM DCFH-DA (2⁣′,7⁣′-dichlorofluorescin diacetate) at 37°C for 60 min. DCFH-DA was then oxidized to DCF (dichlorofluorescein) by ROS in the cell. After washing two times with PBS, the fluorescence intensity in cell lysates was detected by flow cytometry.

### 2.8. Transmission Electron Microscopy (TEM)

Liver tissue was prefixed with 2.5% glutaraldehyde at 4°C for 30 min and then postfixed in 1% osmium tetroxide for 1 h. Then, the tissues were dehydrated with a graded ethanol series from 50% to 100%. Next, the tissues were suspended in propylene oxide twice for 15 min and then soaked in a 1:1 mixture of acetone:epoxy at 40°C for 6 h. The tissues were then covered with pure epoxy resin at 40°C for 4 h. The liver tissues were then sliced and stained with 4% aqueous uranyl acetate and 0.3% lead citrate. After washed with double-distilled water, the ultrastructure of the autophagic vacuoles was observed and captured under a TEM (HT7700, Hitachi).

### 2.9. Reverse Transcription Polymerase Chain Reaction (RT-PCR)

The total RNA was extracted from liver tissues or cultured cell samples according to the manufacturer's procedures (TRIzol reagent, #15596026, Invitrogen, MA, United States). The cDNA was synthesized using a reverse transcriptase kit (#04896866001, Roche, Basel, Switzerland). Quantitative polymerase chain reaction (qPCR) was performed in a real-time system (Bio-Rad) by using SYBR green (#04887352001, Roche). Each qPCR reaction was performed in duplicate. The primer pairs are listed in Table 1.

### 2.10. Western Blot (WB) Analysis

The protein was extracted by using the specific protein lysis buffer (P0013B, Beyotime, China). Protein samples (50 *μ*g) were separated on a 10% SDS-PAGE gel and transferred to a polyvinylidene fluoride (PVDF) membrane. The membranes were blocked with a buffer containing 5% nonfat milk. Then, the membranes were incubated with the indicated primary antibodies: anti-p38 antibody (14064-1-AP, Proteintech), anti-p-p38 (protein p38) antibody (28796-1-AP, Proteintech), anti-FTO antibody (27226-1-AP, Proteintech), anti-Bcl-2 (B-cell lymphoma/leukemia 2) antibody (BA0412, BOSTER), anti-Bax antibody (50599-2-lg, Proteintech), anti-LC3 antibody (81004-1-RR, Proteintech), anti-p62 antibody (A19700, ABclonal), and anti-*β*-actin antibody (66009-1-Ig, Proteintech). The membranes were washed with PBS-Tween 20 and were incubated with the corresponding secondary antibodies for 1 h at room temperature. The expression levels of the target proteins were normalized to *β*-actin.

### 2.11. In Vitro Hypoxia/Reoxygenation (H/R) Model

A human hepatocyte cell line was cultured in Dulbecco's Modified Eagle Medium (DMEM) containing 10% fetal bovine serum (Thermo). The cell culture was placed in an incubator chamber at 37°C with 5% CO_2_ (normal air condition). The H/R model in vitro was established as described in our recently published study [7]. Firstly, the cultured cells were treated with hypoxia for 1 h (1% O_2_, 5% CO_2_, and 94% N_2_). Meanwhile, the serum-free DMEM-F12 medium was used. Subsequently, to perform reoxygenation, the fresh normal maintenance media was used, and the plates were placed under normal conditions. After cultured for indicated times, the cells and medium were collected for further analysis. For FTO inhibitor FB23-2 treatment, the cells were cultured in a normal maintenance medium added with FB23-2 (2 *μ*m; Macklin, F793072, Shanghai, China) and then subjected to H/R stimuli.

### 2.12. Monodansylcadaverine (MDC) Staining

Formation and accumulation of autophagosomes in cells were detected by MDC (Beyotime, C3018S, Nanjing, China) dyes. Following the manufacturer's experimental protocol, 2 × 10^5^ cells were plated in a six-well plate overnight. After different group stimuli, cells were treated with MDC solution at 37°C in the dark for 30 min. Next, the cells were washed with assay buffer, followed by fluorescence microscope observation.

### 2.13. Data Analysis and Statistics

Statistical analysis was performed by using GraphPad Prism software as described in our recently published study [7]. To compare the difference between the two groups, Student's *t*-test (two-tailed, unpaired) was used. For multiple comparisons, a one-way analysis of variance (ANOVA) was used, with the least squares difference (LSD) test to analyze the difference between the two groups. Differences were considered statistically significant at *p* < 0.05. All results were shown as mean ± SD.

## 3. Results

### 3.1. Expression of FTO in Liver I/R at Different Time Points

**Figure 1:**
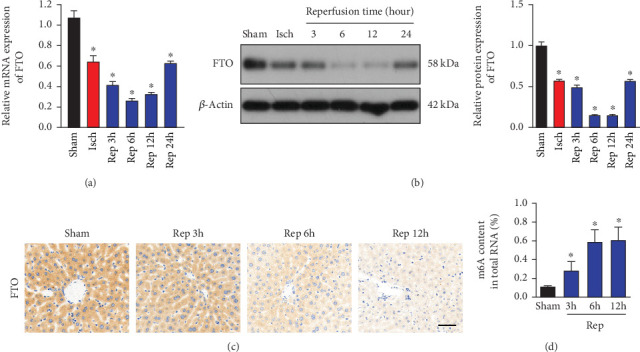
Expression of FTO in different time points of hepatic ischemia-reperfusion injury (IRI). (a) qRT-PCR was used to detect FTO expression at different time points of hepatic IRI. (b) Western blot was used to detect FTO protein levels at different time points of hepatic IRI. (c) The expression of FTO at the protein level was verified by immunohistochemistry staining. Scale bar, 50 *μ*m. (d) Total levels of methylated mRNA (m^6^A) in liver tissues after I/R injury were detected by the m^6^A RNA Methylation Quantification Kit. ⁣^∗^*p* < 0.05 versus Sham; *n* = 5 mice for each group.

The levels of FTO were investigated by using RT-PCR and WB analysis at different hepatic I/R time points. As shown in Figures 1(a) and 1(b), FTO expression in ischemia and different Rep groups was significantly decreased compared with the sham group. Among them, the expression level of FTO was the lowest in the Rep 6 h group, so 6 h were selected for follow-up experiments. Next, the downregulation of FTO in the liver after I/R injury was further verified by immunohistochemistry staining (Figure 1(c)). In contrast, the level of m^6^A-methylated mRNA was increased in the liver of mice after the I/R injury procedure (Figure 1(d)). These results indicated that FTO and related m^6^A demethylation might be involved in the pathogenesis of hepatic I/R injury.

### 3.2. Effect of AAV-FTO on Liver Tissue Injury and Apoptosis

**Figure 2:**
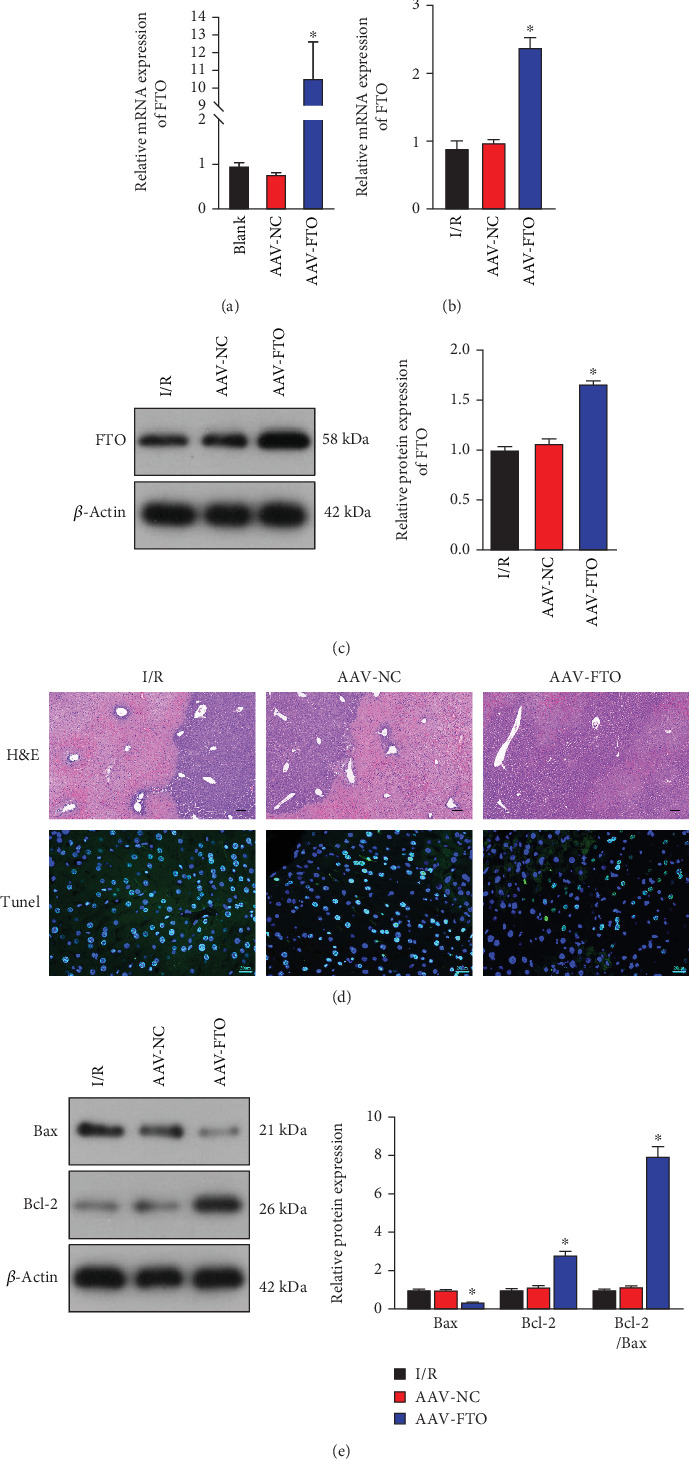
AAV-FTO mitigated hepatic ischemia-reperfusion injury and apoptosis. (a) Transfection efficiency was detected by RT-PCR. (b, c) RT-PCR and western blot analysis of FTO expression. (d) HE and TUNEL assay were used to test liver tissue injury and apoptosis. *n* = 6 mice for each group. Scale bar, 100 *μ*m for HE images; 20 *μ*m for TUNEL images. (e) Bax and Bcl-2 protein expression were detected by western blot. ⁣^∗^*p* < 0.05 versus AAV-NC group; *n* = 3.

We first tested the transfection efficiency of AAV-FTO. As shown in Figure 2(a), compared with the blank group or adeno-associated virus negative control (NC) (AAV-NC) group, the expression of FTO in the AAV-FTO group was significantly increased, indicating successful transfection of AAV-FTO. Subsequently, RT-PCR and WB were used to detect the changes in FTO expression in each group, and the results are shown in Figures 2(b) and 2(c). Compared with the AAV-NC group, FTO expression in the AAV-FTO group was significantly increased. Compared with the AAV-NC group, liver tissue damage (HE staining) and apoptosis (TUNEL staining) were reduced in the AAV-FTO group (Figure 2(d)). We further detected changes in the expression of apoptosis-related proteins Bax and Bcl-2. Compared with the AAV-NC group, the expression of Bax was significantly decreased in the AAV-FTO group, while the level of Bcl-2 was significantly increased (Figure 2(e)).

### 3.3. Effect of AAV-FTO on Liver Tissue Autophagy and p38 Mitogen-Activated Protein Kinase (MAPK) Pathway

**Figure 3:**
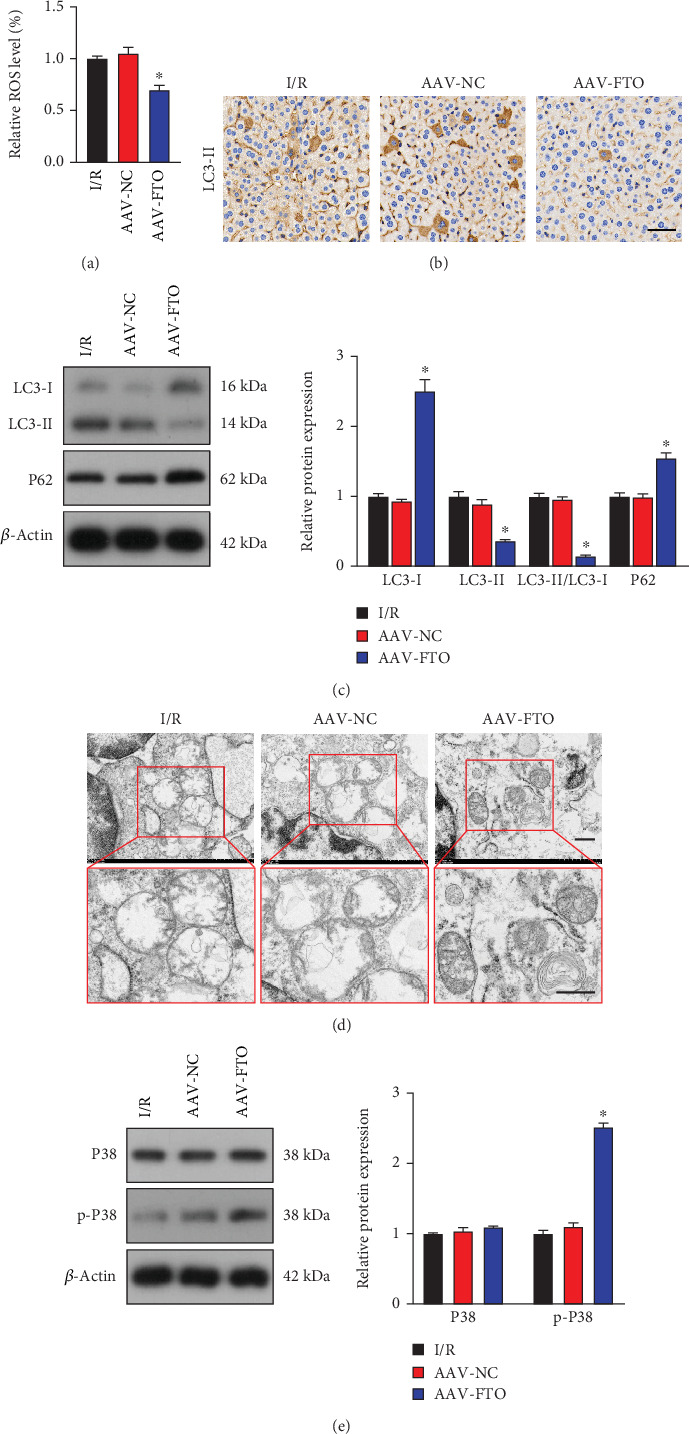
Effect of AAV-FTO on liver tissue autophagy and p38 MAPK pathway. (a) ROS levels were determined by flow cytometry. (b) Immunohistochemical was used to detect the LC3-II expression. Scale bar, 50 *μ*m. (c) Levels of LC3-I, LC3-II, and p62 were tested by western blot. (d) Mitochondrial autophagy was observed by transmission electron microscopy. Scale bar, 5 *μ*m. (e) Western blot analysis of p38 and p-p38 protein expression. ⁣^∗^*p* < 0.05 versus AAV-NC group; *n* = 3.

As shown in Figure 3(a), ROS contents in the AAV-FTO group were decreased compared with those in the AAV-NC group. Immunohistochemistry was used to detect changes in the expression of LC3-II in each group, and the results showed that LC3-II expression levels were reduced in the AAV-FTO group compared to the AAV-NC group (Figure 3(b)). WB was further used for verification, and the results showed that compared with the AAV-NC group, LC3-I and p62 expression levels in the AAV-FTO group were significantly increased, while LC3-II expression levels were significantly decreased (Figure 3(c)). TEM assays were used to observe the characteristics of autophagy. Results showed that excessive and great autophagic vacuoles containing cellular material were observed in the liver after I/R injury. However, compared with the AAV-NC group, autophagic vacuoles were reduced in the AAV-FTO group (Figure 3(d)). To detect the effect of AAV-FTO on the p38 MAPK pathway, a WB was used to detect the key proteins in the p38 MAPK signaling pathway. The results showed that compared with the AAV-NC group, the expression levels of p-p38 in the AAV-FTO group were significantly increased (Figure 3(e)).

### 3.4. FB23-2 Inhibits FTO Demethylation Activity to Aggravate Autophagy in Hepatocytes Upon H/R-Induced Damage

**Figure 4:**
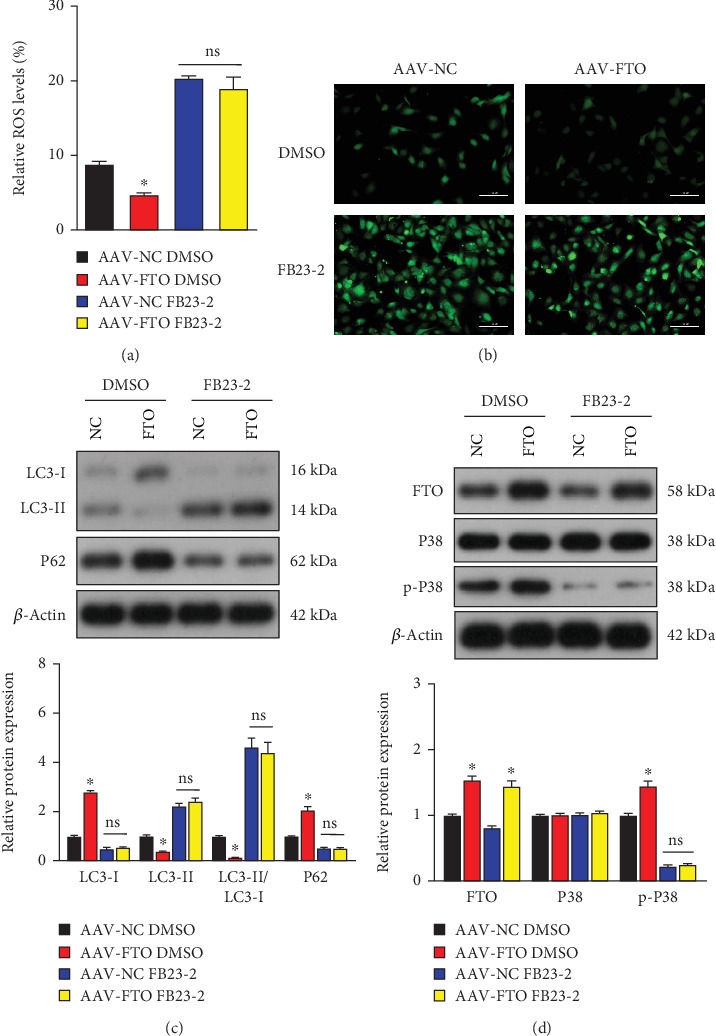
FB23-2 suppresses the demethylation activity of FTO to aggravate hepatocyte autophagy induced by H/R in vitro. Cultured L02 hepatocytes transfected with the indicated plasmids were treated with DMSO or FB23-2 (2 *μ*m). (a) Intracellular ROS levels were determined by flow cytometry. (b) Representative fluorescence images of cells stained with MDC to visualize autophagic vesicles. Scale bar, 100 *μ*m. (c) Protein expression levels of LC3-I, LC3-II, and p62 were detected by western blot. (d) Western blot analysis of FTO, p38, and p-p38 protein expression. ⁣^∗^*p* < 0.05 versus AAV-NC DMSO group; ns, no significance.

Next, we studied the demethylase activity of FTO in regulating hepatocyte autophagy and the p38 MAPK pathway upon H/R-induced damage. Cultured L02 hepatocytes were transfected with FTO overexpression (OE-FTO) or NC plasmids. Then, the hepatocytes were treated with FB23-2 to selectively inhibit FTO's m^6^A demethylase activity [10]. Consistent with the in vivo result, FTO overexpression decreased ROS contents in L02 hepatocytes in response to H/R stimuli. In contrast, FB23-2 significantly increased ROS contents in both NC and OE-FTO groups (Figure 4(a)). Furthermore, MDC staining showed that FTO could decrease the accumulation of autophagic vacuoles compared to the NC group. FB23-2 aggravated cell autophagy and abolished the protective effects of FTO overexpression in L02 hepatocytes in response to H/R damage (Figure 4(b)). Besides, the effect of FTO in regulating autophagy marker proteins and the p38 MAPK signaling pathway was abolished by FB23-2. In dimethylsulfoxide (DMSO) treatment groups, results showed that LC3-I and p62 protein expression levels in the OE-FTO group were significantly increased, while LC3-II expression levels were significantly decreased (Figure 4(c)). Consistently, phosphorylation levels of p-p38 were increased by FTO overexpression in comparison to the NC group (Figure 4(d)). However, these protein expression levels showed no difference between the OE-FTO and NC groups after treatment with FB23-2 (Figures 4(c) and 4(d)). Collectively, our data suggested that the regulating role of FTO on excessive hepatocyte autophagy and the p38 MAPK pathway was dependent on its demethylation activity.

## 4. Discussion

Liver IRI refers to the process of liver tissue injury caused by the interruption of blood flow or oxygen supply and then recovery of liver tissue, which is mainly caused by organ function damage caused by liver resection, transplantation, and other operations [11]. IRI will not only cause direct damage to liver cells but also affect the regeneration ability of liver cells, which is an important factor affecting the success rate of liver surgery and postoperative survival rate. Therefore, it is of great clinical significance to find an effective method to reduce liver IRI [12–14].

FTO is the first protein found to have demethylase activity. It works with m^6^A methyltransferase to regulate m^6^A levels in vivo. Thus, it plays an important regulatory role in human physiological and pathological processes, and its discovery confirms the dynamic and reversible nature of m^6^A modification [15]. In recent years, it has been found that FTO, as a demethylation enzyme, is closely related to cell apoptosis. In a variety of diseases, FTO can reduce mRNA m^6^A levels, promote cell proliferation, and reduce cell apoptosis [16–18]. In this study, our results showed that the expression level of FTO decreased to varying degrees during the occurrence of IRI, which was accompanied by a gradual increase in m^6^A accumulation. These results indicated that FTO-mediated m^6^A demethylation was closely involved in the development of hepatic I/R injury.

At present, the pathways involved in apoptosis mainly include the mitochondrial pathway, death receptor pathway, and endoplasmic reticulum pathway [19], and the mitochondrial pathway is the main pathway of apoptosis. Studies have shown that the Bcl-2 protein family and cysteine aspartic acid–specific protease (caspase family) play an important role in the mitochondrial apoptosis pathway. The Bcl-2 protein family is an important regulatory factor of apoptosis, which can be divided into (1) the Bcl-2 subfamily, containing the BH4 domain, which can inhibit apoptosis [20], and (2) the Bax subfamily and BH3 subfamily, which have the necessary domain BH3 for promoting apoptosis [21]. Bcl-2 gene, the first antiapoptotic gene discovered, mainly exists in the outer membrane of mitochondria and can play antiapoptotic roles through various mechanisms, such as reducing mitochondrial membrane potential, inhibiting the release of cytochrome C (cytC), and blocking the activation of apoptotic protease. Bax protein mostly exists in the cytoplasm in an inactive state and is activated after receiving the apoptotic signal to play a proapoptotic role by destroying the integrity of the mitochondrial membrane and other mechanisms [22, 23]. The Bcl-2 gene and the Bax gene act in opposition to each other, and the ratio between the two proteins is considered to be the key to cell survival [24]. Therefore, in this study, HE staining and TUNEL staining were used to observe the effects of AAV-FTO on liver injury and apoptosis, and the results showed that after increasing FTO expression, liver injury and apoptosis were improved. We further detected the expression levels of Bax and Bcl-2, and the results were still consistent with the above results. It is suggested that AAV-FTO can inhibit the apoptosis of IRI. It has been well recognized that hepatic I/R injury is marked by parenchymal cell death and inflammatory response, and both hepatocytes and inflammatory cells are critically important for the development of liver damage induced by I/R injury [25, 26]. The main purpose of this study is to clarify the role of FTO in regulating hepatocyte damage upon I/R injury. However, the role of FTO in nonparenchymal cells, such as Kupffer cells, in regulating liver damage induced by I/R stimuli needs further research.

Autophagy is a process by which eukaryotic cells use lysosomes to degrade damaged proteins and organelles [27]. Generally, autophagy usually plays a protective role in cells, but excessive autophagy under stress conditions may interrupt homeostasis and even lead to cell death [28]. Autophagy contributes to the pathogenesis of diverse diseases. Importantly, exploring the precise regulation of autophagy, especially how to fine-tune excessive autophagy, might be helpful in developing the therapeutic strategy for diseases. Canonical autophagy is executed under the regulation of autophagy-related genes [29]. LC3 runs through the entire autophagy process and is currently recognized as an autophagy marker [30]. After the synthesis of LC3 protein, the C-terminal 5 peptide is clipped by Atg4, and the glycine residues are exposed to produce cytoplasmic localization of LC3-I. During autophagy, LC3-I is processed and modified by ubiquitin-like systems, including Atg3 and Atg7. Besides, LC3-I is coupled with phosphatidyl ethanolamine (PE) to form LC3-II. LC3-II is localized in the inner and outer membranes of autophagy. In response to stimuli, the autophagosome was interacted with and fused with the lysosome. LC3-II on the outer membrane was cleaved by Atg4 to produce LC3-I recycling. LC3-II on the intima was degraded by lysosome enzymes, resulting in a very low LC3 content in autophagic lysosomes [31, 32]. Therefore, the degree of autophagy can be detected by detecting the expression of LC3. In this study, we detected the contents of ROS, LC3-I, and LC3-II in each group, and the results showed that the expression levels of ROS and LC3-II were decreased and the level of LC3-I was increased in the AAV-FTO group. p62 is a widely studied autophagy substrate, which acts as a bridge between LC3 and polyubiquitination proteins. During the formation of an autophagosome, p62 is selectively wrapped into the autophagosome. In the autophagolysosome, p62 is degraded by proteolytic enzymes [33]. Thus, the autophagy level could be evaluated by WB detection of p62 protein expression. Our results showed that the expression levels of p62 were significantly increased by FTO overexpression, suggesting that FTO could inhibit excessive hepatocyte autophagy upon H/R-induced damage. Besides, our data suggested that the regulating role of FTO on excessive hepatocyte autophagy and the p38 MAPK pathway was dependent on its demethylation activity.

## 5. Conclusion

In conclusion, FTO protects the liver against IRI, which is associated with decreased apoptosis and autophagy. FTO-mediated m^6^A demethylation modification may be an important therapeutic target for hepatic IRI.

## Figures and Tables

**Table 1:** Primer sequences.

**Primer**	**Sequence (5**⁣′**-3**⁣′**)**
FTO-F	TCACCAGGGAGACTGCTATT
FTO-R	GGATCAAAGGATTTCAACGA
GAPDH-F	CCTTCCGTGTTCCTAC
GAPDH-R	GACAACCTGGTCCTCA

## Data Availability

The data used to support the findings of this study are included in the article.
